# Interactions between Phoretic Mites and the Arabian Rhinoceros Beetle, *Oryctes* *agamemnon arabicus*

**DOI:** 10.1673/031.012.12801

**Published:** 2012-11-03

**Authors:** Mohammad Ali Al-Deeb, Sabir Bin Muzaffar, Eyas Mohammad Sharif

**Affiliations:** ^1^ Biology Department, Faculty of Science, UAE University, P.O. Box 17551, Al-Ain, UAE; Tel: 0097137136527; ^2^ AI-Foah Date Farm, Al-Ain, UAE

**Keywords:** date palm tree, *Hypoaspis rhinocerotis*, necromeny, phoresy, *Sancassania*

## Abstract

*Oryctes agamemnon arabicus* (Coleoptera: Scarabaeidae) is one of the main pests on date palm trees in the United Arab Emirates (UAE). Two mite species were found associated with this beetle: *Sancassania* sp. (Acari: Astigmata: Acaridae) and *Hypoaspis rhinocerotis* Oudemans (Acari: Mesostigmata: Laelapidae). *Sancassania* deutonymphs (hypopi) were phoretic on *O. a. arabicus* adults and larvae. However, they were also necromenic, because once the host dies they feed on its carcass. The highest deutonymph load was found in the subelytral space of *O. a. arabicus* adult. The phoretic and necromenic interactions between *Sancassania* sp. and *O. a. arabicus* need to be investigated in more detail. *H. rhinocerotis* was recorded for the first time in UAE. Its role has not yet been studied in the date palm agricultural ecosystem.

## Introduction

Many species of *Oryctes* beetles are important pests of coconut palm (*Cocos nucifera*), oil palm (*Elais guineensis*), and date palm trees (*Phoenix dactylifera*) around the world (Bedford 1980). Adult beetles may reduce fruit yield and kill seedlings and adult trees, resulting in large economic losses. One of the species endemic to the greater Middle Eastern region is *Oryctes agamemnon* (Coleoptera: Scarabaeidae) (Gassouma 1991; Howarth and Gillett 2008; Soltani et al. 2008,). Two other species, *Oryctes rhinoceros* and *Oryctes elegans*, have been found in the region, although their distribution and exact impact on date palm plantations are not known (Gassouma 1991). Mites are known to be associated with these beetles; however, the identity, type of interaction, and the ecology of these associations have not been studied in the United Arab Emirates (UAE), although *Sancassania* sp. (Acari: Astigmata: Acaridae) was reported as a phoretic mite on *O. agamemnon* for the first time in UAE (Al-Deeb and Enan 2010).

Phoresy is a complex symbiotic association that has evolved in many organisms as a result of spatial and temporal isolation of their habitats (OConnor 1982). Phoresy is defined as a form of commensalism facilitating the physical transport of one organism on the body of another, during which time no feeding or reproduction occurs in the phoretic organism (Binns 1982, OConnor 1982). In the most astigmatid mites (Acari: Astigmata), only one of the life stages, the deutonymph (hypopus), exhibits phoretic behavior and has developed morphological adaptations, such as attachment devices, to aid in phoresy. Phoretic associations are particularly diverse in astigmatid mites that have exploited vertebrate and invertebrate hosts for movement between suitable habitats. Phoresy in astigmatid mites has been more explicitly described as migratory movement of mites from natal habitats using superficial attachment to hosts (Houck and OConnor 1991).

Although the function of phoresy is dispersal, phoretic associations can be complex (Binns 1982; OConnor 1982; Houck and OConnor 1991). Many apparently phoretic mites, for example, may use the association for more than dispersal, and some may even derive nutrition from their host. The hypopodes of the *Hemisarcoptes coopermani*, formerly regarded as a phoretic of the beetle *Chilocorus cacti*, have been shown to acquire fluids from the hemolymph of their beetle hosts (Houck and Cohen 1995), suggesting a transition between a phoretic and a truly parasitic association (Houck and Cohen 1995). Polak (1996) reported that phoretic *Macrocheles* mites pierce *Drosophila* integument and ingest haemolymph, affecting survivorship. Although there is a great body of literature on phoretic mites and their beetle hosts, a consensus on the functions and the adaptive significance of phoresy has not been reached.

*Sancassania* (*Caloglyphus*) (Astigmata: Acaridae) are a poorly known, highly diverse, cryptic genus of mites that commonly occur in moist, decaying habitats such as manure, leaf litter, compost, poultry refuse, and fungal fruiting bodies (OConnor 1982; OConnor 2009). Many species of this genus are frequently associated with dung beetles (Coleoptera: Scarabaeidae) and are assumed to be phoretic on the adult beetle (OConnor 1982). However, some species of *Sancassania* remain on the host from the larval stage through the pupa and into the adult stage (Chmielewski and Lipa 1967). Mites of the mesostigmatid family, Laelapidae, may vary from being generalist predators to facultative or even obligate parasites on animals (Krantz 1978). Of particular interest is the diverse genus *Hypoaspis*, which is also found in association with a variety of scarabaeid beetles, because of their potential roles in biological control of some species. Some adult *Hypoaspis* mite species that are associated with dung beetles feed on beetle eggs and are considered important mortality factors for these beetles (Hinckley 1967; Khanjani and Uechermann 2005). The widely varying mitehost associations provide important study models for examining questions in developmental biology and provide possibilities for biological control of pests such as *O. a. arabicus.*

Preliminary observations on *O. a. arabicus* beetles in the UAE suggested that more than one species of mites may be associated with these beetles. In this paper, mite-beetle associations of *O. a. arabicus* from date palm plantations are studied, and the identity, intensity, and distribution patterns of each mite species on *O. a. arabicus* are determined. Also, the spatial distribution of these mites on the body of their beetle hosts is studied. Furthermore, the possible effects of these mites on one another and on their hosts are discussed.

## Materials and Methods

### Insect and mite collection and preparation

From June to September in 2008, 2009, and 2010, live adults of *O. a. arabicus* beetles were randomly collected from infested date palm plantations at Al-Ain (24° 11′ N, 55° 45′ E), Abu Dhabi, UAE. A total of 375 beetles (277 females and 98 males) were either collected in groups using light traps or picked up individually by hand. The main focus of the collection was on *O. a. arabicus* adults, however 20 larvae, which live in the soil, were also collected. Adults and larvae were placed separately in plastic containers for transportation and storage. In the laboratory, sex of the adult beetles was determined, and the associated mites were counted (see the following section), removed using a fine camel hair-brush, and stored in 70% ethanol.

### Mite distribution on host's body

Mites were counted on the head, thorax, legs, subelytral space, membranous wings (alae), abdominal tergites, and abdominal sternites of each beetle under a dissecting microscope. Mite specimens were cleared in lactophenol solution, mounted in Hoyer's medium on microscope slides (Krantz 1978; Evans 1992), and examined under a compound light microscope for identification. *O. a. arabicus* and mite voucher specimens were placed in the collection of the Biology Department at UAE University.

### *Sancassania* sp. necromenic life style

Ten *O. a. arabicus* adults hosting phoretic *Sancassania* deutonymphs were killed by decapitation using a sterile surgical blade. They were kept in sterile plastic containers with dental wicks soaked with distilled water as a moisture source at room temperature (25 ± 5° C).

### Statistical Analysis

Quantitative Parasitology 3.0 software, specifically developed to account for aggregated distributions and to allow distribution-free statistical tests, was used to compare loads (Reiczigel and Rózsa 2005). Mean intensity (mean number of mites per infected beetle), mean abundance (mean number of mites per beetle), and prevalence (proportion of beetles that hosted mites) were quantified following Rózsa et al. (2000). Confidence intervals (at 95% confidence level) for mean intensities were computed using bootstrap techniques with 2000 replications (Rózsa et al. 2000). Exact Confidence intervals (at 95% confidence level) were calculated for prevalence using the Clopper-Pearson method. Mean intensities and abundance of mites on male and female beetles were compared using Bootstrap t-tests, and *p*-values were generated from 2000 replications (Rózsa et al. 2000). Prevalence of mites on males and females was compared using Fisher's Exact Test, with the exact *p-*value reported. Differences in the mean intensity, mean abundance, and prevalence of different mite species found on *O. a. arabicus* were also compared using the same methods. An Index of Discrepancy was calculated to determine aggregated distributions of mites on *O. a. arabicus.* This index is a measure that was introduced by Poulin (1993). It quantifies the difference between the observed parasite distribution and the hypothetical distribution that corresponds to the ideal case where all hosts harbor the same number of parasites. Data were log-transformed prior to conducting one-way ANOVA on the *Sancassania* phoretic load on host body parts. The presented results are the back transformed data. Means were separated by the least square means procedure (LSMEANS) (SAS Institute 2001).

## Results

### Mite distribution on host's body

**Figure 1.**
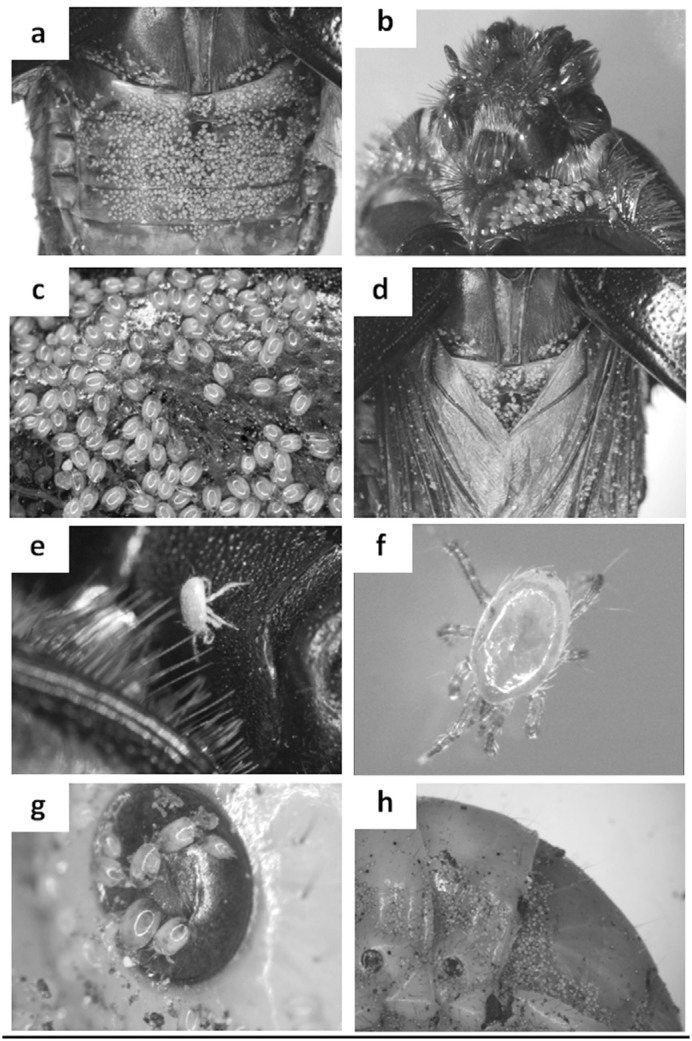
*Sancassnia* sp. deutonymphs and *H. rhinocerotis* on *O*. *agamemnon arabicus* (adult = a–e) (larva = g–h): (a) abdominal tergites; (b) head and prothorax; (c) subelytron; (d) membranous wings; (e, f) *H. rhinocerotis*; **(g) *Sancassnia* sp. deutonymphs on a spiracle of *O. agamemnon arabicus* larva; (h) large number of *Sancassnia* sp. deutonymphs on the body of *O*. *agamemnon arabicus* larva. High quality figures are available online.

**Figure 2.**
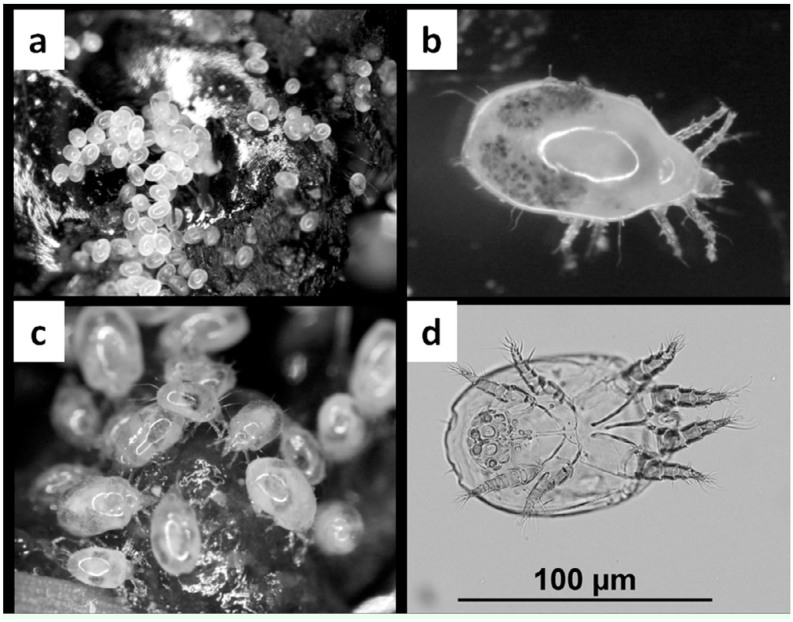
*****Sancassnia* sp.: (a) eggs on *O. agamemnon arabicus* carcass; (b) adult (dorsal view); (c) different life stages feeding on *O.* *agamemnon arabicus* carcass; (d) deutonymph under light compound microscope (×100). High quality figures are available online.

Mite counts data are presented in Table 1. Two mites were found associated with *O. a. arabicus*: *Sancassania* sp. (Acari: Astigmata: Acaridae) (Al-Deeb and Enan 2010) and *Hypoaspis rhinocerotis* Oudemans (Acari: Mesostigmata: Laelapidae) (Costa 1971) (Figures 1 and 2).

*Sancassania* sp. deutonymphs (hypopi) range on *O. a. arabicus* females was bigger than on the males (Table 1). Prevalence of mites on females was significantly lower than on males (Fisher's Exact test, *p* < 0.001; Table 1). However, no significant differences occurred between the mean abundance of *Sancassania* deutonymphs on females and on males (Bootstrap t-test, *t* = -0.614, *p* = 0.5640; Table 1). Additionally, no significant differences were observed between the mean intensity of mites on females and on males (Bootstrap t-test *t* = 0.909, *p* = 0.3860; Table 1). The distribution of *Sancassania* deutonymphs on both female and male beetles was highly aggregated (index of discrepancy values were 0.966 and 0.848, respectively; Table 1). Deutonymphs ranged from 0–7744 individuals per host on *O. a. arabicus* larva. Some tritonymphs were also found on the adults among the phoretic *Sancassania* deutonymphs, but these were not compared statistically because of their small numbers.

**Figure 3.**
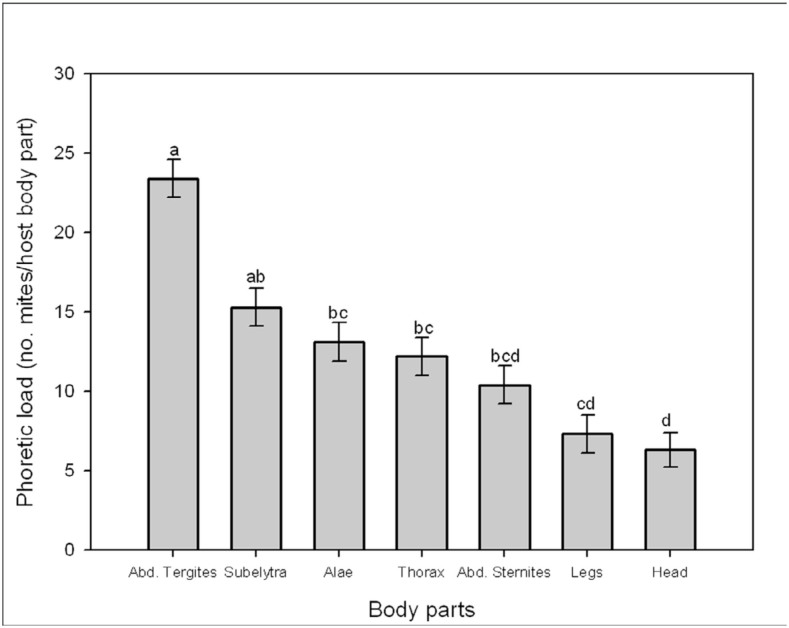
****Phoretic load (Mean ± SE) of *Sancassania* deutonymphs on *O. agamemnon arabicus* adult body parts. Columns labeled with different letters are significantly different (*p* < **0.05) (PROC GLM, LSMEANS, SAS Institute, 2001). Abd = Abdominal; Alae = Membranous wings. High quality figures are available online.

Phoretic load (Mean ± SE) of *Sancassania* deutonymphs on the *O. a. arabicus* body parts is shown in Figure 3. Significant differences in mite loads occurred among the host body parts (one-way ANOVA, *F* = 5.64; df = 6, 275; *p* < 0.0001). The highest load was found on the abdominal tergites (23.4 ± 1.2) and was not significantly different from the load on the subelytra (15.3 ± 1.2) (Table 2). However, the mite load was significantly different from the load on membranous wings (13.1 ± 1.2), thorax (12.2 ± 1.2), abdominal sternites (10.4±1.2), legs (7.3±1.2), and head (6.3±1.1). The least number of deutonymphs was harbored on the head, but was not significantly different from the load on the legs or abdominal sternites. However, the head load differed significantly from the load on the thorax, membranous wings, subelytra, and abdominal tergites (Table 2). Some *O. a.* *arabicus* adults carried a high number of deutonymphs on their bodies. The maximum numbers of deutonymphs recorded on body parts were: subelytra = 1100, membranous wings = 617, abdominal tergites = 1374, thorax = 154, abdominal sternites = 39, head = 38, and legs = 20.

**Table 1.**
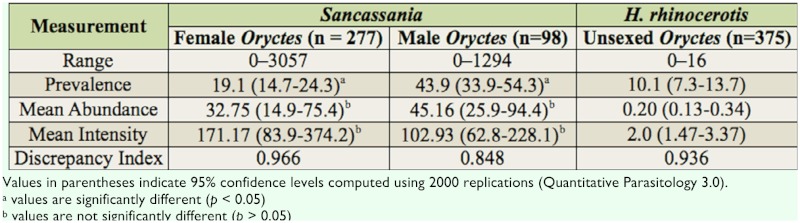
Loads *of Sancassania* deutonymphs and *H. rhinocerotis* mites on *O. a. arabicus.*

*H. rhinocerotis* range (mites per host) varied among *O. a. arabicus* adults. Prevalence of *H. rhinocerotis* was small, and this mite exhibited aggregated distribution on hosts (Table 1).

### *Sancassania* sp. necromenic life style

Adult (Figure 2b) male and female *Sancassania* appeared 3–4 days after the death of the *O. a. arabicus* host. Eggs (Figure 2a) were laid on the body of the dead beetles; they were clearly visible as white spheres on the shiny black exoskeleton of the beetle. Adult and immature *Sancassania* were observed feeding on the soft tissues and fluids of the beetle carcass (Figure 2c). The number of adults increased over time. Phoretic deutonymphs (hypopi) (Figure 2d) were observed aggregating on the underside of the plastic container lid.

**Table 2.**
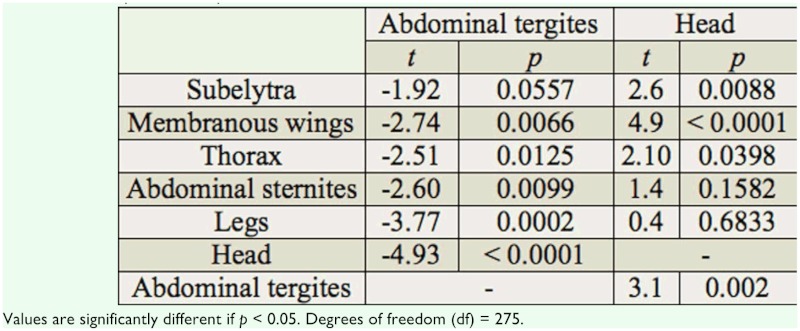
T-test pairwise comparisons of *Sancassania* mite load on *O.* *a. arabicus.*

## Discussion

This study reports the distribution and abundance of *Sancassania* deutonymphs on *O. a. arabicus* beetles. Additionally, sex-specific host choice was recorded. *Sancassania* exhibited niche specialization on the body of the host, with specific areas of the host's body being preferred for attachment.

### Sex-specific prevalence of mites and distribution patterns

Higher prevalence of *Sancassania* on males suggested host sex preference, with the deutonymphs preferring the males over females. Selection of males by *Sancassania* could be due to their size; *Sancassania* deutonymphs could actively choose the larger *O. a. arabicus* males (compared to the females), which provide more space for the attachment of mites. Astigmatid mites of the family Acaridae, such as *Rhizoglyphus echinopus* collected from the scarab beetle *Osmoderma emicola* (Norton 1973), also seem to prefer the males (which are larger than the females). Similarly, Grossman and Smith (2008) reported that the phoretic deutonymphs of the genus *Poecilochirus* (Mesostigmata: Parasitidae) discriminated among adult male *Nicrophorus investigator* beetles based on body size and selected the large males. Additionally, males of *Oryctes* beetles produce pheromones. It has been reported that males of *O. elegans* (Hallett et al. 1995) and *O. rhinoceros* (Rochat et al. 2004) produce aggregation pheromones that help conspecifics locate one another on palm trees. We suspect that these pheromones are utilized by *Sancassania* mites to detect the males of suitable hosts.

Both the mean abundance and mean intensity of *Sancassania* deutonymphs did not differ significantly between males and females of *O. a. arabicus*, suggesting that mites attach to a host regardless of its sex once it is selected.

The distributions of *Sancassania* deutonymphs on both males and females of *O. a. arabicus* were highly aggregated (index of discrepancy values were close to 1; Poulin 1993). Aggregated distributions are characteristic of parasites and may have occurred due to patchy distribution of *Sancassania* habitat in the UAE ecosystem.

### Tritonymphs on beetle hosts and necromeny

Necromeny is an interaction between two species in which the necromenic organism climbs a carrier organism when it is alive and only completes its life cycle after the carrier's death on its cadaver (Schulte 1989). Phoretic *Sancassania* deutonymphs developed into male and female adults a few days after the death of the *O. a. arabicus* host. However, the presence of tritonymphs on living hosts is an uncommon finding, since, it is widely believed, deutonymphs of mites are the only phoretic stage on beetle hosts (Houck and OConnor 1991). It is likely that the tritonymphs documented on living hosts in this study had either developed on them or came from carcasses of dead hosts in the habitat. It is also possible that the host was diseased or aging and had begun giving chemical signals indicating imminent death. Consequently, some of the phoretic deutonymphs aboard may have responded to the signals by resuming their ontogeny. Further studies are required to clarify this aspect of phoretic mite biology on hosts. This study also confirmed the presence of phoretic *Sancassania* deutonymphs on *O. a. arabicus* larvae (Figure 1h). This finding brings into question the nature and extent of this interaction. Larvae of *O. a. arabicus* live in the soil and do not move over long distances in a way that would support phoresy as a mean of dispersal for *Sancassania* deutonymphs. Thus, the interaction between the deutonymphs and the larvae is unlikely to be direct phoresy, since dispersal of the mites is not really achieved. However, if the interaction leads to necromeny, then it is possible that deutonymphs will wait for extended periods for the host to molt into subsequent instars. Soltani et al. (2008) reported that the larval stage of *O. a. arabicus* lasts for ≈ 215 days before they pupate and adults emerge to live for ≈ 65 days. The data from this study suggest that these mites may colonize the larvae of the hosts and may remain present on them for prolonged periods as the host molts and eventually dies, culminating in necromeny subsequent to host death. Necromeny has been documented earlier in *Sancassania* deutonymphs on beetles (Houck and OConnor 1991). The results of the present study showed that the *Sancassania* deutonymphs were also necromenic in the desert ecosystem. It appears that the goal of this phoretic association is not merely for dispersal, by which mites colonize new habitats without losing energy.

### Distribution on the host body

The location of attachment of mites on the body of the beetle is also of potential significance to the host. *Sancassania* mites showed niche specialization on *O. a. arabicus* beetles. The maximum number of phoretic *Sancassania* deutonymphs was found on the abdominal tergites (Figure 1a) and in the subelytral space (Figure 1c), suggesting preference for these niches. Mites on the underside of the elytra or on the abdominal tergites (which are covered completely by the elytra) are protected from the external environment compared to mites found on the other body parts. This protection is very important in the UAE desert climate in which high daytime temperatures (increasing to almost 60° C) and very low moisture levels occur during the spring and summer. Apparently, phoretic *Sancassania* mites select an area on the host body that provides maximum protection from the environment. This finding demonstrates that the spatial distribution of phoretic mites on the host body is mostly nonrandom. However, some mites were attached to other host body parts including the membranous wings, legs, and head. This presence in other locations may be because of the absence of space in favorable sites such as under the elytra.

Some *O. a. arabicus* adults bear very large numbers of phoretic *Sancassania* deutonymphs. In the present study, 3057 phoretic deutonymphs were found on one *O. a. arabicus* female. Phoresy is defined as a dispersal method with low energetic costs to the hosts (Houck and OConnor 1991). We tend to predict that such an extreme mite load is detrimental to the host. Rocha et al. (2009) reported that *Rhyzopertha dominica* adult take-off ability was significantly decreased in the presence of high loads (> 7 mites per host) of phoretic mites. Elzinga and Broce (1988) mentioned that house flies were so burdened with histiostomatid hypopi that they were unable to fly or behave normally. There is no record showing that *Sancassania* deutonymphs feed on their live *O. a. arabicus* host or even cause direct damage to them; however, it is possible that large numbers of them can plug the spiracles of hosts (Figure 1g). Also, their presence on the membranous wings (Figure 1d) and elytra may hamper the host's ability to fly efficiently. In this study, phoretic deutonymphs attached to the host's compound eyes and mouthparts were observed (Figure 1b). Attachment to sensory organs undoubtedly affects vision and could, therefore, also affect flight and feeding behavior. Thus, the observations of *O. a. arabicus* larval infestation by *Sancassania* deutonymphs, necromeny of the adult beetles, and the possible detrimental effects of high mite loads on the adults may suggest that phoresy of *Sancassania* on *O. a. arabicus* beetles could be a transition from commensalism to antagonism. Such transitions have also been documented in other species of mites on beetles, and this could be a mechanism driving the development of parasitism in phoretic mites (Houck and OConnor 1991; Houck and Cohen 1995).

### Host-phoretic ecology

Adults of *O. a. arabicus* are active during spring and summer in UAE. They are present in the date palm plantations from April until the end of September, when their population declines to zero. Soltani et al. (2008) reported that this is a univoltine insect. Because the *Sancassania* mite is phoretic, and subsequently necromenic on *O. a. arabicus*, it is possible that it has synchronized its dispersal and life cycle with its host. In the Astigmata, dispersal is not necessarily directly correlated with habitat quality, but is possibly associated with the life cycle of the host, and is only indirectly correlated with habitat degradation (Houck and OConnor 1991).

The results of this study showed that the phoretic form hypopus does not necessarily develop in response to harsh weather conditions. In the laboratory rearing experiment, *Sancassania* phoretic hypopi were produced, although the rearing settings were not harsh and food was available. Hypopi probably emerged as a result of overcrowding. There might be a threshold population size above which new habitats must be sought. Woodring (1969) stated that dryness, overcrowding, or accumulation of wastes in stock cultures slightly increased the percentage of hypopi of the *Rhizoglyphus echinopns.* However, our finding raises the possibility that hypopi may also develop without any stress. This is supported by the observation of Woodring (1969), who mentioned that on rare occasions, apparently not connected with particularly poor culture conditions, there occurred an unexplained, sudden, tremendous increase in hypopi (up to 25%) in a given stock colony of *R. echinopns.*

*Hypoaspis* is a predatory mite that is found associated with many beetles, especially those in the family Scarabaeidae (Costa 1971). The present study is the first record of the predatory mite *H. rhinocerotis* in UAE. In the present study, this mite was found moving on different body parts of *O. a. arabicus* (Figures 1e, 1f). *H. rhinocerotis* was always found on the ventral side of the body. This is most likely because the underside of the insect gives more protection as a result of having hiding spaces. Most of the time, *H. rhinocerotis* mites were found near the *O. a. arabicus* head on the orange hairs around the palps. We did not observe them feeding on *Sancassania* deutonymphs. However, the predatory behavior of the mites in the genus *Hypoaspis* suggests that the deutonymphs are one of their food supplies while traveling on *O. a. arabicus.* Lesna et al. (1996) mentioned that *Hypoaspis aculeifer* feeds on the bulb mite *Rhizoglyphus robini* and highlighted its use in biological control, as *H. aculeifer* can suppress the bulb mite population to very low levels. Also, *Hypoaspis miles* is an important predatory mite that is used as an agent for biological control of *Bradysia paupera* (Wright and Chambers 1994). More *Hypoaspis* species were recorded in countries neighboring the UAE: *H.* (*Pneumolaelaps*) *azarbaijaniensis* and *H.* (*Hypoaspis*) *polyphyllae* in Iran (Khanjani and Ueckermann 2005; Faraj et al. 2008), and *H. zaheri* and *H. dactylifera* in date palm tree debris in Saudi Arabia (Fouly and Al-Rehiayani 2010). Specific data on the effect of *H. rhinocerotis* on *O. a. arabicus* in UAE are not available, and more research is warranted.

In conclusion, *Sancassania* is phoretic on *O. a. arabicus*; however, this interaction is apparently not mere phoresy, as it involves necromeny. When the host dies, the phoretic deutonymphs ontogeny continues, and the different life stages feed on the soft tissues of the host carcass. Most of the deutonymphs are found on the abdominal tergites, which are covered by the elytra. The presence of *H. rhinocerotis* indicates the likelihood of finding other species in UAE. Some of these mites may participate in the natural biological control of *O. a. arabicus.* More extensive studies on the ecology and the interactions of *Sancassania*, *H. rhinocerotis*, and *O. a. arabicus* are needed.
